# A Granulocyte-Specific Protein S100A12 as a Potential Prognostic Factor Affecting Aggressiveness of Therapy in Patients with Juvenile Idiopathic Arthritis

**DOI:** 10.1155/2018/5349837

**Published:** 2018-10-22

**Authors:** Krzysztof Orczyk, Elzbieta Smolewska

**Affiliations:** Department of Pediatric Cardiology and Rheumatology, Medical University of Lodz, Sporna 36/50, 91-738 Lodz, Poland

## Abstract

**Background:**

Defining new prognostic biomarkers has become one of the most promising perspectives for the long-term care of patients with juvenile idiopathic arthritis (JIA). The new efficient indicators of disease activity and potential response to treatment are crucial in establishing new therapeutic plans in accordance with the “treat to target” strategy. One of the most studied proteins is called S100A12, which is an alarmin specific for granulocytes, considered as a marker of their activity.

**Materials and Methods:**

Study involved 80 patients diagnosed with JIA. Children with systemic subtype were not included in the study. In 53 cases, blood samples were obtained in two time points. Results from the study group were compared to 29 age- and sex-matched healthy individuals.

**Results:**

Serum S100A12 levels were higher in JIA than in healthy controls at the study baseline (11.67 ± 6.59 vs. 6.01 ± 2.33 ng/ml). There were no significant differences in S100A12 values between assessed subtypes of JIA. The highest concentrations were observed in patients within a disease flare. S100A12 levels were not dependent from using glucocorticosteroids. The studied protein appeared to be an efficient biomarker for JIA patients: 100% specificity as a diagnostic marker (cut-off level: 10.73 ng/ml) and 100% sensitivity as an indicator of exacerbations within a 3-month observation (cut-off level: 5.48 ng/ml).

**Conclusions:**

S100A12 may become an important factor influencing decisions on aggressiveness of JIA therapy. Further elaboration on the clinical algorithm is necessary for that protein to be included in everyday practice.

## 1. Background

### 1.1. Juvenile Idiopathic Arthritis

Juvenile idiopathic arthritis (JIA) is a heterogenous group of arthritides affecting children under 16 years of age. It should be suspected in patients with symptoms persisting for at least 6 weeks without an apparent cause [1]. Essential part of the diagnostic process involves eliminating arthropathies caused by the known etiological factors included in the so-called “list of exclusions” [2]. A lack of unequivocal clinical features or laboratory findings frequently results in the delay of the final diagnosis. According to Aoust et al. [3], JIA was suspected only in 37% of patients from the disease onset. The remaining patients were presenting symptoms for 3 months (on average) before being diagnosed with JIA.

### 1.2. Disease Activity

JIA patients may considerably differ from each other in terms of the severity of their disease. In the cohort study of 609 children reported by Guzman et al. [4], who analyzed disease activity and patients' quality of life within a 5-year observation, four distinct types of JIA course were defined: mild (which is the most common, affecting 43.8% of patients), moderate (35.6%), severe controlled (9.0%), and severe persisting (11.5%). Taking into consideration the aforementioned classification, the adequate assessment of disease activity and response to treatment is crucial for effective long-term care of JIA patients.

Juvenile arthritis disease activity score (JADAS) is a widely used tool created to evaluate disease activity [5] in everyday practice of a pediatric rheumatologist. It contains four parameters: (1) physician global assessment, (2) patient or parent global assessment, (3) normalized value of erythrocyte sedimentation rate (ESR), and (4) active joint count. In the recent study, the version involving 27 joints (JADAS27) was utilized due to its simplicity and strong correlation with the entire assessment of 71 joints [6].

### 1.3. Treat to Target

The “treat to target” concept of therapy, which is recently applied in rheumatoid arthritis patients, has already attracted interest of pediatric rheumatologists as well [7]. According to this innovative approach, the aggressiveness of treatment should be associated with the therapeutic goal, which may be determined as clinical remission off medication or, if it does not seem achievable, as at least minimal disease activity [8].

Although the effectiveness of early aggressive therapy has been supported by the study of Wallace et al. [9], it may increase the risk of overtreatment in patients, who would have responded to less intensive and, by extension, less toxic therapy [10]. Ravelli et al. did not report significant difference in frequency of remission in patients with oligoarticular JIA treated with intraarticular glucocorticosteroids (GCS) in monotherapy (remission in 32%) or combined with methotrexate (remission in 37%) [11]. Blazina et al. [10] underscored the necessity of utilizing prognostic biomarkers in making decisions on aggressiveness of treatment.

### 1.4. “Classic” and New Biomarkers

“Classic” serological markers, which include rheumatoid factor (RF), anticyclic citrullinated peptide autoantibodies (ACPA), and antinuclear antibodies (ANA) [12], are helpful in assigning patients into separate JIA subtypes or estimating risk of comorbidities (such as uveitis). However, they do not provide sufficient data on disease activity or response to treatment to predict the future course of the disease. Therefore, more and more potential markers are studied in order to facilitate a long-term care of JIA patients. In a systematic review presented by Gohar et al. [13], there were 68 biomarkers evaluated in systemic JIA. Fifty of them were assessed by only a single research group. The authors postulated consolidation of findings and further validation of markers which are already identified. Thus, the recent study involved one of the most studied and the most promising biomarkers, which is called S100A12.

### 1.5. S100A12

S100A12, also known as calgranulin C, is one of the calcium-binding S100 family proteins. This alarmin is specific for granulocytes; therefore, it may be considered as an indicator of their activity [14]. It presents strong chemotactic activity as a ligand binding receptor for advanced glycation end products (RAGE) [15, 16]. S100A12 participates in recruitment of inflammatory cells in murine models [17]. Previous findings reflect overexpression of S100A12 in inflamed tissues in adult patients with inflammatory bowel diseases, psoriatic arthritis, and rheumatoid arthritis [18, 19]. Moreover, serum concentration of S100A12 is considered as a marker of disease activity in children with Kawasaki disease [20].

Being an indicator of granulocytes' activity, S100A12 may appear useful as a marker of disease activity in JIA patients. The S100A12 level in synovial fluid was 10 times higher than in serum in a study published by Foell et al. [21], which demonstrated its potential as a reliable marker of local inflammation. Furthermore, serum concentration of that protein was higher in patients with active arthritis than in children with stable remission. Baseline S100A12 levels were elevated in patients with good response to intraarticular GCS. Serum S100A12 concentrations were also increased in children who exacerbated within 6 months after measurement. Similar findings were reported by Yamasaki et al., who observed higher S100A12 levels in patients who were unable to maintain remission for 2 years [22]. Additionally, S100A12 was the best isolated biomarker for prognosing disease flares in the further validation performed by Gerss et al. [23].

The most recent study by Gohar et al. [24] confirmed the relation between S100A12 concentration and the effectiveness of therapy. Patients with a good response to methotrexate and tumor necrosis factor (TNF) inhibitors had higher baseline S100A12 levels than treatment-refractory children. Combining methotrexate with systemic GCS did not affect the marker values. The authors suggested further elaboration on the ideal algorithm involving S100A12 in therapeutic decisions.

The present study was conducted in order to evaluate whether it is reliable to measure S100A12 serum levels in JIA patients in everyday practice of the pediatric rheumatologist. The objective of the study was to assess the clinical significance of S100A12 as a diagnostic biomarker of JIA and prognostic indicator of increasing disease activity within a 3-month observation.

## 2. Materials and Methods

The study involved 80 patients diagnosed with JIA who were admitted to the Department of Pediatric Cardiology and Rheumatology, Medical University of Lodz, Poland, between January 2017 and February 2018. In 53 cases, blood samples were collected in two time points (with an average interval of 102.4 ± 26.0 days) in order to evaluate dynamics of serum S100A12 concentrations. Patients diagnosed with systemic JIA were excluded from the study because of its distinct pathogenesis. Results from the study group were compared to the control group containing 29 age- and sex-matched individuals who were referred to the Department due to functional cardiovascular system dysfunction.

Patients' records were comprehensively reviewed in order to build a database containing age at diagnosis and age at evaluation; JIA subtype according to International League of Associations for Rheumatology (ILAR) classification [2]; reason of admission (fresh diagnosis of JIA, disease flare, continuation of biological treatment, and check-up visit); active joint count; JADAS27 value and disease activity level determined using cut-off levels proposed by Consolaro et al. [25, 26] (patients with enthesitis-related arthritis were assessed with criteria for oligo- and polyarticular JIAs, depending on the active joint count); current therapy, including disease-modifying antirheumatic drugs, intraarticular and systemic GCS, and biological agents.

After obtaining blood samples, the following laboratory tests were ordered: complete blood count, ESR, C reactive protein (CRP), and “classic” serological markers (RF, ACPA, and ANA). Collected blood samples were also stored in −80°C in order to determine serum concentrations of S100A12 protein using ELISA Kit SEB080Hu (Cloud-Clone, China).

All statistical calculations were carried out using Statistica 13.1 software (Statsoft Polska, Krakow, Poland). The values were expressed as mean ± standard deviation (SD). The Shapiro-Wilk test was performed to assess the normality of continuous variables. Spearman's rank correlation coefficients were calculated for variables not normally distributed. The Mann-Whitney *U* test and Kruskall-Wallis test were utilized for group comparisons. The receiver operating characteristic (ROC) curve was drawn for S100A12, and the area under the curve (AUC) was computed to assess its diagnostic and prognostic significance. 95% confidence intervals (CI) were calculated for sensitivity, specificity, and AUC. *P* values lower than 0.05 were considered significant.

The study was approved by the Bioethics Committee of the Medical University of Lodz (approval no. RNN/31/17/KE).

## 3. Results

Characteristics of the study group are presented in Table 1. Serum S100A12 levels were significantly elevated in JIA patients at first time point (11.67 ± 6.59 ng/ml, *P* < 0.001) when compared to the control group (6.01 ± 2.33 ng/ml). Concentrations measured in the study group at second time point were also higher (7.45 ± 4.81 ng/ml) than in healthy individuals, but the difference did not reach statistical significance (*P* = 0.40). S100A12 values were independent from age at diagnosis (*P* = 0.899), age at the study baseline (*P* = 0.768), and sex (*P* = 0.534) of patients. They were also not related to “classic” serological markers. Moreover, levels of the protein did not differ (*P* = 0.121) between assessed subtypes of JIA (RF-positive polyarthritis was excluded from this part of analysis due to the small sample size).

**Figure 1:**
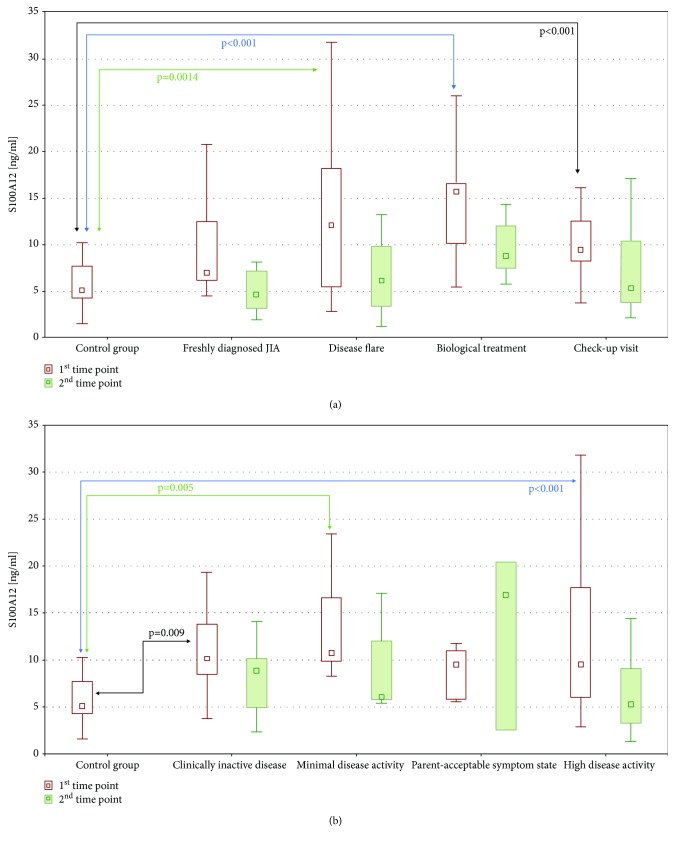
S100A12 levels depending on (a) reason of admission and (b) disease activity level. *P* values were presented only for statistically significant comparisons. JIA: juvenile idiopathic arthritis.

Serum S100A12 concentrations were increased in JIA patients regardless of the reason of admission (Figure 1(a)). The highest values of S100A12 were observed in patients with disease flare, but the differences between subgroups of JIA patients were not statistically significant. S100A12 concentrations were significantly correlated with CRP (*r* = 0.473, *P* < 0.001) and ESR (*r* = 0.353, *P* < 0.001) values. In terms of the disease activity level (based on JADAS27), the most increased S100A12 concentrations were noted in patients with high disease activity (Figure 1(b)). However, that difference was not significant as well.

**Figure 2:**
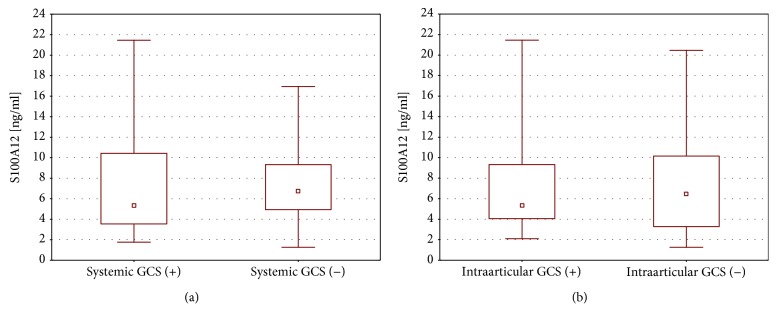
S100A12 levels at 2^nd^ time point depending on (a) systemic GCS intake within the observation period and (b) intraarticular GCS injection at 1^st^ time point. GCS: glucocorticosteroids.

Apart from disease-modifying antirheumatic drugs (all patients in the study group were treated with methotrexate), forty-three JIA patients (53.8%) were using systemic GCS at first time point. Moreover, in twenty-four children (30.0%), intraarticular GCS were necessary during the first hospitalization within the study. However, S100A12 levels did not differ significantly (Figure 2) depending on usage of systemic or intraarticular GCS (*P* = 0.766 and *P* = 1.00, respectively). Nine patients were also treated with biological agents, but the significant difference in S100A12 levels was not observed (*P* = 0.0853).

Considering values noted at second time point, S100A12 levels increased in 14 patients (average change: 3.63 ± 3.50 ng/ml). Concurrent elevation of JADAS27 was observed in 2 (14.3%) patients. Concentrations of the marker decreased in 39 patients (average change: 7.23 ± 5.49 ng/ml). Simultaneous decline of JADAS27 was noted in 26 (66.7%) children. Differences in S100A12 levels between both time points were statistically significant (*P* < 0.001).

**Figure 3:**
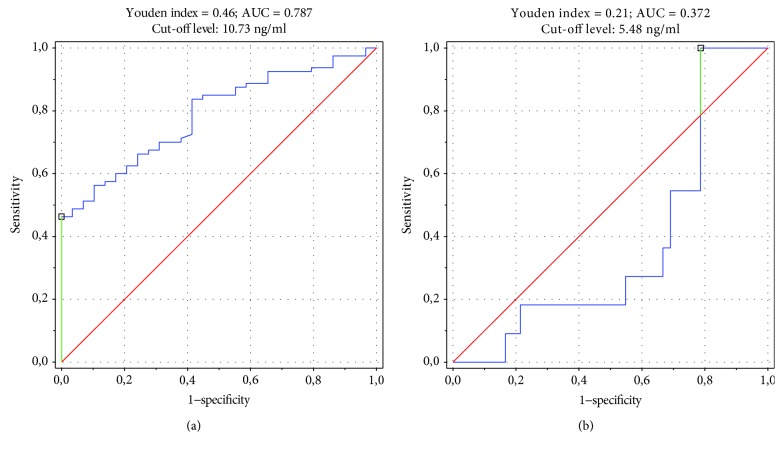
Receiver operating characteristic curve for S100A12 as (a) JIA diagnostic biomarker and (b) disease flare prognostic biomarker. AUC: area under the curve; JIA: juvenile idiopathic arthritis.

S100A12 had 100.0% specificity (95% CI: 88.1%—upper limit is not applicable) and 46.3% sensitivity (95% CI: 35.0–57.8%) as a diagnostic JIA biomarker for cut-off level 10.73 ng/ml with AUC 0.787 (95% CI: 0.701–0.873). The ROC curve is illustrated in Figure 3(a). S100A12 was also assessed as a potential prognostic marker for predicting exacerbations of the disease. It was characterized with 100.0% sensitivity (95% CI: 71.5%—upper limit is not applicable), 21.4% specificity (95% CI: 10.3–36.8%), and AUC 0.372 (95% CI: 0.203–0.542) as an indicator of disease flare within a 3-month observation (Figure 3(b)).

## 4. Discussion

Personalized treatment involving the “treat to target” strategy opens a new chapter in the therapeutic approach in JIA patients. Effectiveness of such therapy can be maximized by reliable assessment of disease activity, both in patients presenting symptoms and in children achieving clinical remission on medication. One of the potential diagnostic and prognostic biomarkers, S100A12 protein, was included in our study in order to evaluate its clinical significance.

Serum S100A12 levels were significantly higher in JIA patients when compared to healthy individuals. These results are consistent with findings of Foell et al. [21] and Bojko [27]. Unlike Yamasaki et al. [22], we did not observe significant differences in S100A12 values between included JIA subtypes. However, the remarkable specificity of S100A12 (100% for cut-off value 10.73 ng/ml) suggests potential utilization of that biomarker in differential diagnosis in patients suspected of JIA.

Serum S100A12 concentrations were noticeably increased in patients with high disease activity. The hundred-percent sensitivity in predicting disease flare within a 3-month observation (cut-off value 5.48 ng/ml) supports findings of the previous study by Gerss et al. [23]. As reported by Gohar et al. [24], S100A12 values were independent from GCS intake, which makes that protein a reliable prognostic marker in patients who need to use them. In our study group, more than a half of JIA patients were taking systemic GCS at the study baseline.

Although S100A12 is postulated as an indicator of treatment response [24], decrease of its serum concentration was related to the decline in the JADAS27 value only in 66.7% of patients. Therefore, it should not be considered as an isolated marker of the effectiveness of therapy. On the other hand, Giancane et al. [28] stated that the pain should be considered as a direct cause and main indication for treatment in JIA patients. Older age at diagnosis and longer disease duration are the risk factors of lower efficacy in reduction of patients' symptoms [29]. Measuring the JADAS value on the day of the check-up visit may not be the most informative indicator of patients' well-being between the assessments, which may have influenced the evaluation of the relation between S100A12 and disease activity. Smartphone applications should be elaborated on in order to get the better feedback from the patient [28, 30].

The main limitation of the study was the considerable diversity of the study group. A reliable assessment of effectiveness of treatment and measurements of potential biomarker values should be performed in a possibly homogenous group of patients independent from factors influencing the results. Additionally, the findings from the study group should be compared with patients with arthritides other than JIA in order to evaluate the usefulness of S100A12 in differential diagnosis.

## 5. Conclusions

Including S100A12 protein in everyday practice of the pediatric rheumatologists might be helpful in making therapeutic decisions. Escalation of aggressiveness of therapy in the right group of patients may potentially reduce the frequency of disease flares. Setting the clinical algorithm of ordering the S100A12 test is challenging until it becomes widely accessible in clinical laboratories.

## Figures and Tables

**Table 1:** General characteristics of the study group.

	1^st^ time point (*n* = 80)	2^nd^ time point (*n* = 53)
Female, *n* (%)	54 (67.5%)	36 (67.9%)
Age at diagnosis (years)	7.75 ± 4.27	7.57 ± 4.41
Age at evaluation (years)	10.40 ± 4.38	10.28 ± 4.58
Reason of admission, *n* (%)		
Fresh diagnosis of JIA	16 (20.0%)	—
Disease flare	26 (32.5%)	11 (20.8%)
Continuation of biological treatment	9 (11.25%)	12 (22.6%)
Check-up visit	29 (36.25%)	30 (56.6%)
JIA subtypes, *n* (%)		
Oligoarticular JIA	54 (67.5%)	35 (66.0%)
RF-negative polyarticular JIA	11 (13.75%)	9 (17.0%)
RF-positive polyarticular JIA	1 (1.25%)	1 (1.9%)
ERA	14 (17.5%)	8 (15.1%)
Clinical and laboratory features		
Fever on admission, *n* (%)	9 (11.3%)	0 (0.0%)
CRP > 5 mg/l, *n* (%)	13 (16.3%)	6 (11.3%)
ESR > 20 mm/h, *n* (%)	16 (20.0%)	4 (7.5%)
Disease activity level (according to JADAS27), *n* (%)		
Clinically inactive disease	19 (23.75%)	16 (30.2%)
Minimal disease activity	7 (8.75%)	12 (22.65%)
Parent-acceptable symptom state	5 (6.25%)	6 (11.3%)
High disease activity	49 (61.25%)	19 (35.85%)

JIA: juvenile idiopathic arthritis; RF: rheumatoid factor; ERA: enthesitis-related arthritis; JADAS27: juvenile arthritis disease activity score 27-joint reduced count.

## Data Availability

The data used to support the findings of this study are included within the article.
